# Data on swan arrival, departure, and population size on the Asadokoro tidal flat, Aomori Prefecture, Japan, from 1956 to 2010

**DOI:** 10.1016/j.dib.2021.106825

**Published:** 2021-02-02

**Authors:** Masaki Ogata, Takeshi Mitsuya, Yoshiyuki Tanaka

**Affiliations:** a Hiranai Town Board of Education, Shimotsuki 12-1, Kominato, Hiranai, Aomori 039-3321, Japan; b Hiranai Society for Swan Protection, Shimotsuki 12-1, Kominato, Hiranai, Aomori 039-3321, Japan; c Department of Life and Environmental Science, Hachinohe Institute of Technology, Ohbiraki 88-1 Myo, Hachinohe, Aomori 031-8501, Japan

**Keywords:** Citizen, *Cygnus cygnus*, Migratory bird, Population record, Bird watching, Elementary school student, Long term

## Abstract

The arrival and departure dates and the daily maximum populations of migrating swans (*Cygnus cygnus*) on the Asadokoro tidal flat, Hiranai town, Aomori Prefecture, Japan, were recorded by elementary school students for more than 50 years between 1956 and 2010. The Asadokoro tidal flat, which lies along the coast of Mutsu Bay, has been designated a National Special Natural Monument, known as “The swans of Kominato and their migration grounds.” This long history of observation unfortunately came to an end with the closure of the elementary school in 2012. If analyzed together with data on environmental factors, such as temperature changes or the effects of avian influenza, this dataset could provide a potentially valuable source of information, and consequently, future secondary use of the data is anticipated.

## Specifications Table

**Table:** 

Subject	Biological Sciences
Specific subject area	Ecology, Behavior
Type of data	Table
How data were acquired	Visual observations and counting
Data format	Raw
Parameters for data collection	Long-term continuous observations were considered. Elementary school students conducted these observations systematically as an extracurricular activity over more than 50 years.
Description of data collection	Elementary school students undertook observations and recording to the best of their ability.
Data source location	Hiranai Town, Aomori Prefecture Japan WGS84, Latitude: 40.93894166, Longitude: 140.97181666
Data accessibility	Mendeley data [9] Published: 28 Jan 2021|Version 1| https://doi.org/10.17632/g9tcw92bgy.1

## Value of the Data

• Data on fluctuations in migratory bird populations were collected over a period of over 50 years, based on observations performed by elementary school students.
• The study site and swans are designated as special natural monuments of Japan [1]. It is argued that a reduction in the number of swans in Hiranai town may have led to a decline in the tourism value of the local area. The data obtained can be used to influence administrative decisions at national and regional levels.
• The data are also of potential value for analyzing changes in the number of swans flying to this site in relation to environmental factors, such as temperature [2], avian influenza [3,4], and human activities [5,6]. They may also prove useful for estimating the impact of migratory birds on the surrounding environment, such as vegetation [7,8]. For example, the arrival of migratory birds at this site has a substantial effect on the distribution of seagrass [1].

## Data Description

The data presented herein relate to the size of the swan population on the Asadokoro tidal flat and the dates on which the swans arrived at and departed from the study site. Table 1 shows the dates from 1956 to 2009 on which the first swans arrived at the Asadokoro tidal flat in autumn, together with the number of individuals observed on each date, whereas Table 2 shows the dates from 1957 to 2010 (mainly in spring) when the last swans departed from the site, together with the number of individuals observed on each date. Table 3 presents the monthly maximum numbers of observed swans per day from 1960 to 2010, and Table 4 summarizes the timing of data acquisition. The raw data file was deposited in Mendeley data (http://dx.doi.org/10.17632/g9tcw92bgy.1)

**Table 1:** The date on which the first swans arrived at Asadokoro tidal flat and the number of individuals. The breakdown of adults and juveniles is also indicated.

Year	Month	Day	Number of individuals	Remarks
1956	10	23	6	
1957	10	19	8	
1958	10	15	8	
1959	10	15	8	
1960	10	20	18	
1961	11	2	8	
1962	10	10	5	
1963	11	2	5	
1964	11	2	5	
1965	10	31	4	
1966	10	21	3	
1967	11	11	6	Adult 6
1968	11	9	1	
1969	11	7	4	Adult 3, Young 1
1970	11	1	3	Adult 3
1971	11	8	7	Adult 2, Young 5
1972	10	26	4	
1973	11	1	6	Adult 2, Young 4
1974	11	1	4	Adult 2, Young 2
1975	11	2	11	
1976	10	14	6	Adult 2, Young 4
1977	10	19	2	Adult 2
1978	10	28	7	Adult 5, Young 2
1979	10	29	13	Adult 4, Young 9
1980	11	10	13	
1981	10	20	24	Adult 20, Young 4
1982	11	4	2	Adult 2
1983	10	19	20	Adult 16, Young 4
1984	10	7	3	Adult 3
1985	10	15	3	Adult 3
1986	10	16	7	Adult 5, Young 2
1987	10	15	10	Adult 10
1988	10	17	16	Adult 16
1989	10	21	4	Adult 4
1990	10	11	3	Adult 3
1991	10	15	2	Adult 2
1992	10	15	30	Adult 26, Young 4
1993	10	16	5	Adult 4, Young 1
1994	10	21	18	Adult 14, Young 4
1995	10	24	7	Adult 7
1996	10	12	3	Adult 3
1997	10	24	2	Adult 2
1998	10	22	18	Adult 14, Young 4
1999	10	20	27	Adult 24, Young 3
2000	10	20	52	Adult 38, Young 14
2001	10	20	17	Adult 9, Young 8
2002	10	21	27	Adult 24, Young 3
2003	10	20	70	Adult 58, Young 12
2004	10	20	22	Adult 22
2005	10	24	135	Adult 103, Young 32
2006	10	24	36	Adult 33, Young 3
2007	10	25	96	Adult 88, Young 8
2008	10	31	39	Adult 30, Young 9
2009	10	20	1	Adult 1

**Table 2:** The date on which the last swans departed from Asadokoro tidal flat and the number of Individuals. The breakdown of adults and juveniles is also indicated.

Year	Month	Day	Number of individuals	Remarks
1957	3	15	1	Record by Mr. Wada
1958	2	11	28	Record by Mr. Wada
1959	2	10	3	Record by Mr. Wada
1960	5	24	1	Record by Mr. Wada
1961	6	18	35	
1962	3	10	2	
1963	3	21	12	
1964	4	26	4	
1965	4	12	6	
1966	4	4	7	
1967	4	8	4	Adult 4
1968	4	8	5	
1969	4	13	7	
1970	4	6	4	
1971	4	6	4	Adult 1, Young 3
1972	4	2	5	Adult 2, Young 3
1973	4	16	23	
1974	4	20	1	Adult 1
1975	4	18	1	Adult 1
1976	4	7	38	
1977	4	12	2	Young 2
1978	4	13	19	Adult 6, Young 13
1979	4	5	13	Adult 8, Young 5
1980	4	20	3	Adult 1, Young 2
1981	4	18	6	Adult 4, Young 2
1982	4	19	23	Adult 23
1983	4	23	3	Adult 3
1984	5	14	2	Adult 1, Young 1
1985	5	4	2	Adult 1, Young 1
1986	4	28	4	Adult 4
1987	5	13	3	Adult 1, Young 2
1988	4	27	1	Young 1
1989	4	18	3	Adult 3
1990	4	19	2	Adult 2
1991	5	17	3	Adult 1, Young 2
1992	5	7	1	Young 1
1993	4	26	1	Adult 1
1994	4	27	4	Adult 2, Young 2
1995	4	28	3	Adult 3
1996	5	20	6	Adult 5, Young 1
1997	5	1	5	Adult 2, Young 3
1998	4	30	1	Adult 1
1999	5	1	5	Adult 4, Young 1
2000	4	28	4	Adult 4
2001	4	27	6	Adult 6
2002	4	22	7	Adult 6, Young 1
2003	4	30	3	Adult 2, Young 1
2004	4	23	3	Adult 3
2005	5	2	8	Adult 8
2006	4	24	9	Adult 7, Young 2
2007	4	18	11	Adult 8, Young 3
2008	4	16	10	Adult 4, Young 6
2009	4	27	2	Adult 2
2010	3	18	1	Adult 1

**Table 3:** Monthly maximum numbers of swan observed each day. The underlined numbers denote the highest population counts during each fiscal year, including the following April.

Fiscal year	Sep	Oct	Nov	Dec	Jan	Feb	Mar	Apr	May
1960	0	39	120	180	200	210	120	0	
1961	0	0	60	172	420	304	18	0	
1962	0	8	70	160	300	265	50	0	
1963	0	0	58	170	260	606	105	4	
1964	0	0	68	174	243	616	203	4	
1965	0	4	94	166	703	1,058	91	7	
1966	0	3	24	206	623	447	225	24	
1967	0	0	61	77	450	789	379	8	
1968	0	0	27	64	613	636	282	24	
1969	0	0	29	352	1,123	834	326	18	
1970	0	0	76	401	630	460	316	34	
1971	0	0	66	482	545	411	259	5	
1972	0	34	256	446	508	438	207	7	
1973	0	0	261	597	514	472	343	54	
1974	0	0	489	333	526	551	269	32	
1975	0	0	174	433	372	434	360	38	
1976	0	6	151	514	666	614	345	21	
1977	1	24	251	501	553	698	485	81	
1978	0	11	150	364	677	532	444	75	
1979	0	23	250	466	625	638	470	131	
1980	0	3	325	644	644	634	509	113	
1981	0	157	195	426	754	679	623	94	
1982	0	0	327	351	742	690	618	153	
1983	0	71	182	447	745	744	585	292	11
1984	0	126	223	354	722	758	688	149	2
1985	0	125	206	503	751	720	682	105	
1986	0	80	182	612	806	795	503	188	10
1987	0	33	233	470	626	738	714	117	
1988	0	55	195	815	950	705	435	104	
1989	0	101	180	447	745	684	486	57	
1990	0	58	392	542	950	788	507	83	10
1991	0	148	273	511	688	733	657	283	6
1992	0	30	204	372	567	527	485	75	
1993	0	156	158	403	878	827	484	132	
1994	0	66	167	422	583	704	472	104	
1995	0	10	158	361	649	654	440	154	21
1996	0	115	202	394	670	704	557	114	5
1997	0	40	240	418	659	690	540	48	
1998	0	49	240	376	591	625	592	118	5
1999	0	45	166	402	476	517	465	110	
2000	0	76	165	320	462	411	360	141	
2001	0	68	279	270	317	390	334	65	
2002	0	297	145	325	432	406	380	99	
2003	0	347	154	219	350	408	303	78	
2004	0	48	203	277	315	302	310	63	8
2005	0	135	128	411	339	365	291	31	
2006	0	39	117	219	324	249	199	20	
2007	0	96	201	170	344	317	256	35	
2008	0	39	46	138	243	203	186	0	
2009	0	2	13	113	154	190	5	0

**Table 4:** Timing of observations. ○ = research was conducted. R = rest, no research. N.D. = no data (although the original data were lost, conditions appear to have been similar to those in the previous and subsequent years.

	Survey time zone		Weekend		
Fiscal year	AM	PM	AM	PM	New Year
1966	8:00	3:00	○	○	○
1967	8:00	3:00	○	○	○
1968	8:00	3:00	○	○	○
1969	N. D.	N. D.	N. D.	N. D.	N. D.
1970	8:00	3:00	○	○	○
1971	8:00	3:00	○	○	○
1972	N. D.	N. D.	N. D.	N. D.	N. D.
1973	8:00	3:00	○	○	○
1974	8:00	3:00	○	○	○
1975	N. D.	N. D.	N. D.	N. D.	N. D.
1976	8:00	3:00	○	R	○
1977	8:00	3:00	○	R	○
1978	8:00	3:00	○	○	○
1979	8:00	3:00	○	○	○
1980	8:00	3:00	○	○	○
1981	8:00	3:00	○	○	○
1982	8:00	3:00	○	○	○
1983	8:00	3:00	○	○	○
1984	8:00	3:00	○	○	○
1985	8:00	3:00	○	○	○
1986	8:00	3:00	○	○	○
1987	8:00	3:00	○	○	○
1988	8:00	3:00	○	○	○
1989	8:00	3:00	○	○	○
1990	8:00	3:00	R	R	R (29 Dec -3 Jan)
1991	8:00	3:00	R	R	R (29 Dec -3 Jan)
1992	8:00	3:00	R	R	R (28 Dec -5 Jan)
1993	8:00	3:00	R	R	R (29 Dec -3 Jan)
1994	8:00	3:00	R	R	R (29 Dec -3 Jan)
1995	8:00	3:00	R	R	R (28 Dec -7 Jan)
1996	8:00	3:00	R	R	R (28 Dec -5 Jan)
1997	8:00	3:00	R	R	R (29 Dec -4 Jan)
1998	8:00	3:00	R	R	R (29 Dec -3 Jan)
1999	8:00	3:00	R	R	R (29 Dec -3 Jan)
2000	8:00	3:00	R	R	R (28 Dec -3 Jan)
2001	8:00	3:00	R	R	R (29 Dec -3 Jan)
2002	10:00	3:00	R	R	R (28 Dec -5 Jan)
2003	10:00	3:00	R	R	R (29 Dec -4 Jan)
2004	10:00	3:00	R	R	R (29 Dec -3 Jan)
2005	10:00	3:00	R	R	R (29 Dec -3 Jan)
2006	10:00	3:00	R	R	R (29 Dec -3 Jan)
2007	10:00	3:00	R	R	R (29 Dec -3 Jan)
2008	10:00	R	R	R	R (27 Dec -4 Jan)
2009	10:00	R	R	R	R (18 Dec -18 Jan)

## Experimental Design, Materials and Methods

**Fig. 1:**
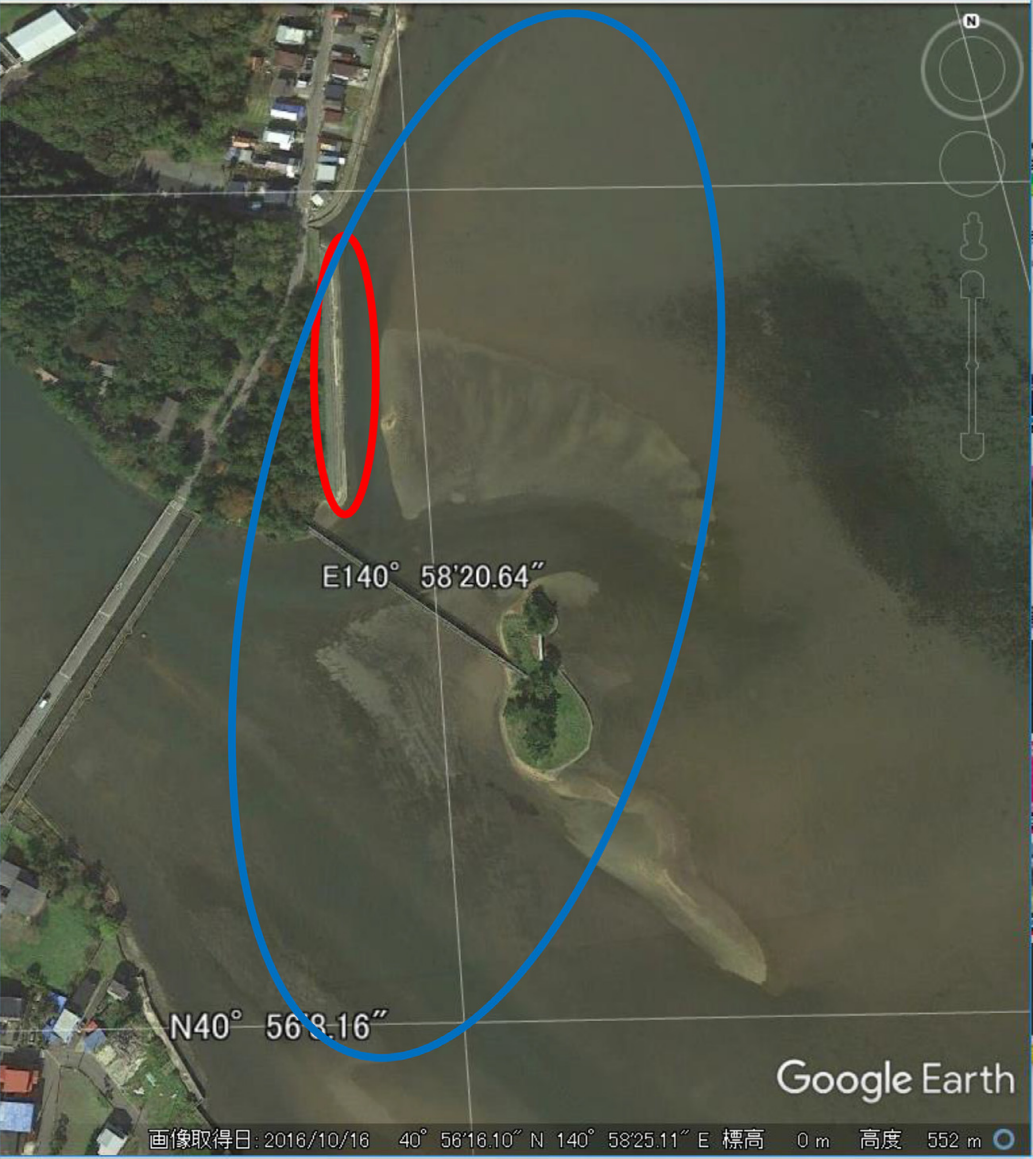
Study site. The figure was modified from a Google Earth Pro image. The red ellipse indicates the area around which the elementary school students walked to observe the swans. The blue ellipse indicates the approximate area in which the swans under observation were present.

In each of the years from 1956 to 2010, observations and recording of swan populations were carried out almost daily at Asadokoro tidal flat (Fig. 1). Each year, students from the Asadokoro Elementary School, which was adjacent to the survey site, formed a team of dozens of individuals under the guidance of teachers, and conducted daily observations in rotation. Teachers gave the students clear instructions to count the number of swans visible from the designated observation location (Fig. 1) within the prescribed time (Table 4). If possible, they also tried to distinguish between adult and young birds. Adult birds are white, whereas young birds are gray, making them easy to distinguish. In this paper, we present a summary of the observational data relating to the day when the first swans arrived (Table 1), the day on which the last swans departed from the tidal flat (Table 2), and the maximum number of swans observed per day in each month of the observational period (Table 3). Tables 1 to 3 show compilations of the raw data extracted by teachers and students. The timing of observations is given in Table 4. As some of the raw data are missing, it is not possible to provide the timing of observations prior to 1965; however, it is assumed that data were acquired according to schedules similar to those used after 1966, as summarized in Tables 1 to 3. From 1966 to 1989, observations were carried out from the time of arrival of the first swans to their departure, including during the year-end and New Year holidays and weekends. However, after 1990, observations were suspended for approximately one week during the year-end and New Year holidays, and observations were not made on Saturdays or Sundays. In 2009, observations were conducted only on Tuesdays and Thursdays, as contact with wild birds at this time was discouraged owing to the death of swans from avian influenza in Aomori Prefecture in 2008 [3,4]. However, during arrival and departure periods, intensive observations were carried out regardless of the day of the week.

Observations were conducted twice daily, at approximately 8:00 am before the start of classes and at approximately 3:00 pm after classes had finished. From 2002 until the final year of observations in 2010, the timing of the morning survey was changed to approximately 10:00 am, coinciding with the morning break. Observations during each survey period were conducted for approximately 20 min. Of the two daily counts, the one yielding the largest number of individuals was taken as the population number for that particular day. Given that swans differ considerably from other migratory birds that fly to this area with respect to size and color, it is assumed that the elementary school students are unlikely to have confused the swans with other species. However, it is conceivable that they may not have been able to distinguish between the whooper swan *Cygnus cygnus* and the tundra swan *C. columbianus*. Nevertheless, the results of a recent survey conducted by the Hiranai Society for Swan Protection have indicated that most of the swans observed in this area are whooper swans (Mitsuya et al., unpublished data).

Although the survey was conducted for the final time in the fiscal year 2010, the data for that year were excluded from the dataset presented herein, owing to the lack of certain population data. In March 2012, Asadokoro Elementary School closed down, and the monitoring of swans that had continued for more than 50 years ended. To date, there has been no resumption of similar monitoring.

## Ethics Statement

Not applicable.

## CRediT Author Statement

**Masaki Ogata:** Resources, Writing - Original Draft; **Takeshi Mitsuya:** Resources, Data Curation; **Yoshiyuki Tanaka:** Data Curation, Funding acquisition, Writing - Review & Editing.

## Declaration of Competing Interest

The authors declare that they have no competing financial interests or personal relationships that influenced or could be perceived to have influenced the work reported in this article.
